# Which factors influence liver stiffness measured by real-time two dimensional shear wave elastography in patients on maintenance hemodialysis?

**DOI:** 10.3325/cmj.2021.62.34

**Published:** 2021-02

**Authors:** Danijela Zjačić Puljiz, Ivana Kristina Delić Jukić, Marko Puljiz, Lučana Vicelić Čutura, Iva Jerčić Martinić-Cezar, Dorotea Božić, Kristian Podrug, Željko Puljiz

**Affiliations:** 1 Department of Nephrology, Split University Hospital, Split, Croatia; 2 Department of Emergency Medicine, Split Center for Emergency Medicine, Split, Croatia; 3 Department of Hematology, Split University Hospital, Split, Croatia; 4 Department of Transfusion Medicine, Split University Hospital, Split, Croatia; 5 Department of Gastroenterology and Hepatology, Split University Hospital, Split, Croatia

## Abstract

**Aim:**

To evaluate liver stiffness (LS) by real-time two-dimensional shear wave elastography (RT 2D-SWE) and to assess its correlation with the mean arterial pressure (MAP) in patients on maintenance hemodialysis (MHD). The secondary aim was to identify biological and biochemical parameters associated with elevated LS.

**Methods:**

This study enrolled patients treated with MHD in the Split University Hospital from December 2017 through February 2018. LS was measured after a HD session using RT 2D-SWE. Mean arterial pressure was measured before RT-2D-SWE was performed.

**Results:**

The study enrolled 47 patients with the mean ± standard deviation age of 68.48 ± 14.33 years. Arterial hypertension was diagnosed in 70.2% of patients. Liver stiffness >7 kPa, suggesting clinically relevant fibrosis, was found in 59.5% of patients. Arterial pressure was significantly correlated with LS (ρ = 0.38, *P* = 0.008). C-reactive protein (ρ = 0.548, *P* = 0.023), parathyroid hormone (ρ = 0.507, *P* = 0.038), and total bilirubin (ρ = 0.423, *P* = 0.020) were correlated with elevated LS.

**Conclusion:**

Mean arterial pressure is correlated with increased LS in patients on MHD. Our results emphasize the importance of proper regulation of arterial blood pressure and indicate that LS should always be interpreted in combination with laboratory parameters. Further prospective studies with larger series are needed.

Chronic kidney disease (CKD) is an important public health problem. The number of CKD patients in the world has increased from approximately 10 000 patients in 1973 to 703 243 in 2015 (1,2). All CKD stages and end stage renal disease (ESRD) are associated with high morbidity, increased health care utilization, and mortality (3). Patients on long-term maintenance hemodialysis (MHD) have an increased risk for hepatitis B and C viral infection and chronic liver inflammation. Acute or chronic liver inflammation is frequently followed by a development of liver fibrosis (LF) (4-6). The clinical presentation of LF differs from that of cirrhosis. Mild LF, as well as early stage liver cirrhosis, are asymptomatic in most patients, which points to the importance of an early diagnosis. Appropriate staging of LF is important for prognosis and for therapeutic decision-making.

Percutaneous liver biopsy is still the gold standard for the evaluation and staging of LF (7). It is an invasive procedure with substantial limitations and very serious potential side effects, primarily, post procedural bleeding. The risk is even higher in dialysis patients who have a bleeding tendency due to platelet dysfunction caused by uremic state. Not less important, liver biopsy carries the risk of pneumothorax and hematothorax (8,9). Another limitation is the possibility of a significant sampling error, since the biopsy specimen represents only 1/50 000 the size of the liver parenchyma (10,11). Due to these limitations, many non-invasive tools for the assessment of LF have been recently investigated, showing promising results (11,12). The most frequently applied novel methods, using ultrasound waves for tissue elasticity measurement, are transient elastography and two-dimensional real-time shear wave elastography (RT-2D-SWE) (13,14). RT 2D-SWE is a fast, quantitative method for assessing LF by measuring liver stiffness (LS) in real time. Acoustic push pulses induce shear waves, and their speed is observed as color image in the region of interest (ROI). Velocity (m/s) is determined by measuring the waves passing through the examined tissue. These values are converted into tissue elasticity expressed in kilopascals (Young’s module) (15). 2D-SWE has demonstrated high resolution and excellent reproducibility for assessing LF (16-19).

LS can be affected by different parameters. LS values are elevated in healthy people with BMI>30 kg/m^2^, and in patients with viral hepatitis, primary biliary cholangitis, alcoholic liver disease, and cholestatic liver diseases independent of the degree of fibrosis (20-23). LS correlates significantly with portal pressure and arterial pressure (24-27), and is affected by volume overload: either due to heart failure or liver failure with ascites (11,28). To avoid volume overload, LS measurement should be performed following a hemodialysis (HD) session (29-31). It remains unexplored to what extend blood pressure contributes to LS (25). Little is known about biological and biochemical factors associated with LS changes in ESRD patients on MHD. To the best of our knowledge, most such studies have been conducted on patients with underlying liver disease (32,33). Only a few have focused on factors that influence LS (measured by transient elastography) and fibrosis in HD population (28). In dialysis patients, active chronic inflammation possibly affects the liver and changes LS (34). Another pathophysiological mechanism could be chronic hepatic congestion with subsequent idiopathic noncirrhotic portal hypertension (35). This study aims to investigate the correlation between the mean arterial pressure (MAP) and LS in patients on MHD. The secondary aim was to identify biological and biochemical parameters correlated with elevated LS. Finally, we hypothesized that MAP influenced LS and that C-reactive protein (CRP), parathyroid hormone (PTH), and total bilirubin correlate with the LS value.

## PATIENTS AND METHODS

This single-center study was conducted in the Department of Nephrology and Dialysis of Split University Hospital Center from December 2017 through February 2018. The study enrolled 47 patients older than 18 years treated with MHD due to ESRD for at least 3 months. The exclusion criteria were applied according to EFSUMB 2017 guidelines and included active malignant disease, acute infection, congestive heart failure, and biliary obstruction as revealed by dilated bile ducts and biochemical signs of cholestasis (17). None of the included patients had serological markers of hepatitis B or C, acute hepatitis, history of excessive alcohol abuse (more than 20 g of alcohol per day), or a history of gastrointestinal surgery. Obese patients (BMI>30 kg/m^2^), patients with metabolic disease, autoimmune liver disease, or a history of liver steatosis caused by drug treatment (eg, estrogen, amiodaron, methotrexate, and high dose estrogen) were excluded as well.

The following parameters were collected: general information, age, sex, height (cm), weight (kg), body mass index (BMI), Kt/v, and urea reduction rate (URR). Data on comorbid conditions (diabetes, atrial fibrillation, arterial hypertension) were obtained from medical history. BMI was determined as weight (kg) divided by height (m) squared. Blood samples were taken before a HD session, prior to heparin administration. Laboratory tests performed are shown in Table 1.

**Table 1:** Laboratory data of study population, N = 47

	Total (n = 47)	Men (n = 32)	Women (n = 15)	*P* value
Leukocytes (10^9^/L)	6.45 ± 1.51	6.1 ± 1.46	6.16 ± 1.62	0.623
Hemoglobin (g/L)	115 ± 11.44	115.91 ± 11.47	113.12 ± 11.54	0.459
Platelets ( × 10^9^/L)	211 ± 68.2	216.28 ± 69.49	200.8 ± 66.82	0.471
Glucose (mmol/L)	6.54 ± 2.71	6. 6 ± 2.64	6. 64 ± 2.94	0.845
Bilirubin (μmol/L)	10.89 ± 9.7	11.49 ± 1.7	9.6 ± 2.8	0.395
Aspartate transaminase (U/L)	16.80 ± 8.63	16.19 ± 5.59	18.11 ± 13.1	0.594
Alanine transaminase (U/L)	15.09 ± 6.99	16 ± 7.5	13.3 ± 5.4	0.148
Alkaline phosphatase (U/L)	89.30 ± 48.55	85.75 ± 38.7	96.93 ± 65.8	0.548
Proteins (g/L)	68.17 ± 4.78	68.5 ± 4.96	67.47 ± 4.45	0.48
Albumin (g/L)	39.66 ± 2.98	39.44 ± 2.72	40.13 ± 3.5	0.506
Creatinine (before hemodialysis) (μmol/L)	855 ± 206.43	877.8 ± 190.2	809.3 ± 237.6	0.338
Creatinine (after hemodialysis) (μmol/L)	329 ± 105.31	330.5 ± 111.2	33.4 ± 93.8	0.901
Urea (before hemodialysis) (mmol/L)	23.99 ± 5.97	24.63 ± 5.99	22.62 ± 5.89	0.287
Urea (after hemodialysis) (mmol/L)	6.97 ± 2.35	7.69 ± 2.73	6.42 ± 1.85	0.071
Triglycerides (mmol/L)	2.40 ± 1.78	2.07 ± 1.19	3.11 ± 2.54	0.151
Cholesterol (mmol/L)	4.15 ± 1.1	3.89 ± 0.82	4.69 ± 1.18	0.2
High-density lipoprotein (mmol/L)	0.98 ± 0.27	0.99 ± 0.27	1.01 ± 0.30	0.8
Low-density lipoprotein (mmol/L)	2.27 ± 0.75	2.17 ± 0.70	2.44 ± 0.78	0.27
C-reactive protein (mg/L)	10.72 ± 30.28	10.85 ± 34.9	15.8 ± 31.6	0.629
Ferritin (μg/L)	394.72 ± 218.42	393.4 ± 243.1	424.6 ± 199.1	0.667
Iron (μmol/L)	11.96 ± 4.41	11.43 ± 4.9	12.53 ± 3.1	0.362
Parathyroid hormone (pmol/L)	45.65 ± 44.52	48.3 ± 43.1	41.03 ± 49.5	0.63
Calcium (mmol/L)	2.28 ± 0.14	2.31 ± 0.14	2.21 ± 0.12	0.15
Phosphorus (mmol/L)	1.64 ± 0.59	1.7 ± 0.62	1.53 ± 0.5	0.331

Systolic and diastolic arterial pressures were measured before RT-2D-SWE with the patients in the supine position. MAP was calculated as follows: systolic arterial pressure +2 × diastolic arterial pressure/3.

The patients received three to five hours of hemodialysis twice or three times per week. Ultrafiltration was evaluated volumetrically by the HD machine, depending on patients’ dry weight (ultrafiltration volume was from one to four liters per procedure). The temperature of the dialysis bath ranged from 35.5 °C to 36.6 °C. The bath contained 138-145 mmol/L of sodium, 2 mmol/L of potassium, 32-35 mmol/L of bicarbonates, and 1.25-1.75 mmol/L of calcium. Low flux polysulfone dialyzers were exerted.

LS was measured with Toshiba Aplio 500, Platinum Series Ultrasound system (Canon Medical Systems USA, Inc, Tustin, CA, USA).

RT-2D-SWE was performed approximately 30 minutes after HD, following at least 3 hours of fasting. LS measurement values were expressed in kilopascals. The cut-off value for detecting LF was LS>7 kPa (36,37). The patients were placed in the supine position with the right arm behind the head. The measurement was performed through the intercostal spaces during forced inspiration (17). ROI was placed between a minimum of 2 cm and a maximum of 6 cm beneath the liver capsule (17). Ten measurements were performed and were considered reliable if IQR/median of 10 measurements was <30%. The examinations with no successful quantification after more than 10 attempts were considered failures and were not used. All examinations were conducted by an experienced physician who performed at least one hundred procedures per year.

The study group was stratified according to nutritional status, BMI<or >23 kg/m^2^, following the International Society of Renal Nutrition and Metabolism Panel recommendations, which state that for patients on dialysis, BMI<23 kg/m^2^ is a protein-energy wasting criterion (38-40).

The study protocol was approved by the Ethics Committee of the University Hospital Split (2181-147-01/06/M.S.-17-2). All procedures conducted are in accordance with the 1964 Helsinki declaration and its later amendments. The participants were informed about the study aim and gave informed consent before recruitment.

### Statistical analysis

The sample size was estimated with G*power 3.1.9.7 software for Windows with Cohen classification for effect size (ρ 0.5), with α 0.05 and power (1-β) of 0.95, indicating that it was necessary to include at least 42 participants. Quantitative variables are summarized using descriptive statistics. Normality of distribution was tested with the one-sample Kolmogorov-Smirnov test. The results are expressed as mean ± standard deviation. Differences between the groups (male vs female, BMI<vs >23 kg/m^2^) were tested with the *t* test for independent data. The correlations between LS value and other variables were assessed with the Pearson (ρ) correlation coefficient. Multiple regression analysis including the variables that were shown to be significant was used to analyze the contribution of each variable to the change in LS. The level of significance was set at *P* < 0.05. Statistical analysis was performed with IBM SPSS Statistics for Windows, version 22.0. (IBM Corp., Armonk, NY, USA).

## RESULTS

### Study population

The study enrolled 47 patients (68.1% men). The mean age was 68.48 ± 14.33 years. The most common etiology of ESRD was chronic nephritis. There was no significant difference in LS mean between men and women (*P* = 0.987). No significant difference in LS (*P* = 0.274), MAP (*P* = 0.899), age (*P* = 0.871), and other analyzed laboratory data was found between the groups with BMI<and >23 kg/m^2^. Baseline demographic data are summarized in Table 2. There was no significant difference in any of the analyzed laboratory data between men and women (Table 1).

**Table 2:** Demographic, anthropometric, and end stage renal disease (ESRD) characteristics of the study population*

	Demographic and anthropometric characteristics			
	total (47)	men (n = 32)	women (n = 15)	*P*
Age (years)	68.48 ± 14.33	69.37 ± 14.4	66.6 ± 14.4	0.544
Body mass index	24.28 ± 3.54	23.8 ± 2.92	25. 1 ± 4.6	0.351
Liver stiffness	7.70 ± 2.30	7.71 ± 2.21	7.70 ± 2.57	0.987
Mean arterial pressure	78.8 ± 15.07	88.6 ± 13.2	82.0 ± 14.5	0.146
Sex, n (%)		32 (68.1)	15 (31.9)	
Diabetes mellitus, n (%)	14 (29.8)			
Arterial hypertension, n (%)	33 (70.2)			
Atrial fibrillation, n (%)	3 (6.4)			
Smoking, n (%)	7 (14.9)			

*ESRD characteristics: 76.6% patients had unknown (chronic nephritis); 4.3% had polycistic kidney disease; 2.1% had kidney hypoplasia; 8.5% had diabetic nephropathy; 4.3% had membranoproliferative glomerulonephritis; 2.1% had nephroangiosclerosis; and 2.1% had vesicoureteral reflux.

### Correlation analysis

**Figure 1:**
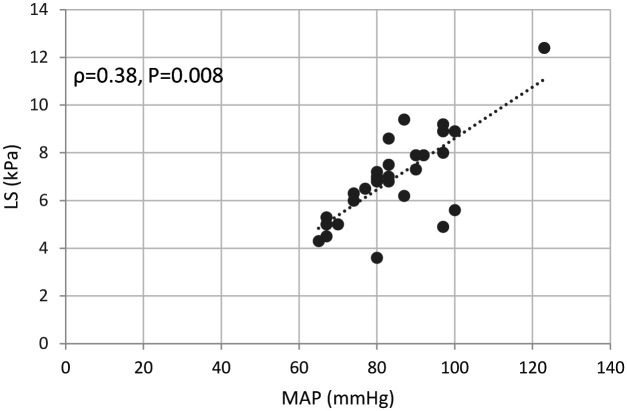
Correlation between mean arterial pressure (MAP) and liver stiffness (LS). ρ –Pearson correlation coefficient.

**Figure 2:**
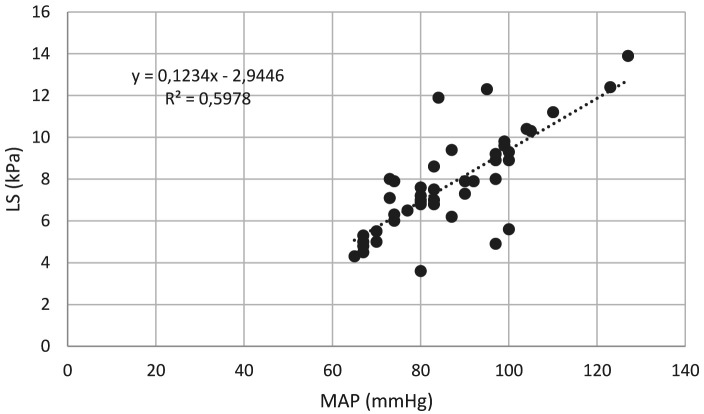
Regression analysis of contribution of mean arterial pressure (MAP) to liver stiffness (LS)

LS measurement >7 kPa, suggesting clinically relevant fibrosis, was found in 59.5% of patients. When patients were stratified according to nutritional status (BMI) (Table 3), 36.1% of patients had BMI<23. In this group, a significant correlation between LS and CRP (ρ = 0.548, *P* = 0.023) and LS and PTH (ρ = 0.507, *P* = 0.038) was found. In the group with BMI>23, a significant correlation was found between LS and total bilirubin (ρ = 0.423, *P* = 0.020). In the group with LS>7 kPa (n = 28), total bilirubin (ρ = 0.515,*P* = 0.005) and PTH (ρ = 0.436, *P* = 0.02) were significantly correlated with LS (Table 4). Arterial blood pressure was significantly correlated with LS (ρ = 0.38, *P* = 0.008) (Figure 1). Multiple regression analysis showed arterial pressure to be an independent predictor of LS (Figure 2).

**Table 3:** Pearson correlation (ρ) analysis of liver stiffness (LS) values and various biochemical parameters in patients stratified according to the body mass index (BMI)

	BMI<23 (n = 17)		BMI>23 (n = 30)	
Parameters	ρ	*P*	ρ	*P*
Leukocytes (10^9^/L)	-0.156	0.594	-0.001	0.996
Hemoglobin (g/L)	-0.22	0.396	-0.069	0.717
Platelets ( × 10^9^/L)	0.286	0.266	-0.07	0.972
Glucose (mmol/L)	-0.178	0.495	-0.72	0.705
Bilirubin (μmol/L)	0.023	0.93	0.423	0.020
Aspartate transaminase (U/L)	-0.019	0.941	0.217	0.249
Alanine transaminase (U/L)	-0.18	0.49	0.196	0.299
Alkaline phosphatase (U/L)	0.12	0.964	-0.083	0.661
Proteins (g/L)	-0.168	0.518	-0.185	0.328
Albumin (g/L)	-0.205	0.43	-0.199	0.292
Creatinine (before hemodialysis) (μmol/L)	0.21	0.418	-0.356	0.054
Creatinine (after hemodialysis) (μmol/L)	0.479	0.61	-0.343	0.068
Urea (before hemodialysis) (mmol/L)	-0.069	0.793	-0.26	0.166
Urea (after hemodialysis) (mmol/L)	0.321	0.226	0.003	0.989
Triglycerides (mmol/L)	-0.281	0.275	-0.78	0.682
Cholesterol (mmol/L)	-0.339	0.183	0.085	0.654
High-density lipoprotein (mmol/L)	0.038	0.884	0.122	0.522
Low-density lipoprotein (mmol/L)	-0.111	0.672	0.318	0.087
C-reactive protein (mg/l)	0.548	0.023	-0.078	0.680
Ferritin (μg/L)	0.18	0.49	0.144	0.448
Iron (μmol/L)	-0.222	0.391	0.204	0.280
Parathyroid hormone (pmol/l)	0.507	0.038	0.141	0.458
Calcium (mmol/L)	0.094	0.721	0.204	0.280
Phosphorus (mmol/L)	0.465	0.6	0.044	0.819
Kt/v	-0.291	0.257	-0.061	0.748
Urea reduction rate	-0.384	0.129	-0.054	0.776

**Table 4:** Pearson correlation analysis of liver stiffness (LS) values and biological and biochemical parameters in patients with end stage renal disease stratified according to liver stiffness value

	LS<7 kPa (n = 19)		LS>7 kPa (n = 28)	
Parameters	ρ	*P*	ρ	*P*
Leukocytes (10^9^/L)	-0.269	0.297	0.292	0.148
Hemoglobin (g/L)	0.175	0.475	0.069	0.728
Platelets ( × 10^9^/L)	0.088	0.721	-0.101	0.609
Glucose (mmol/L)	0.025	0.399	-0.195	0.319
Bilirubin (μmol/L)	0.341	0.153	0.515	0.005
Aspartate transaminase (U/L)	0.352	0.140	0.006	0.976
Alanine transaminase (U/L)	0.043	0.863	-0.117	0.552
Alkaline phosphatase (U/L)	0.019	0.938	0.267	0.170
Proteins (g/L)	0.013	0.957	-0.251	0.198
Albumin (g/L)	-0.246	0.310	-0.161	0.412
Creatinine(before hemodialysis) (μmol/L)	-0.209	0.390	-0.252	0.196
Urea (before hemodialysis) (mmol/L)	-0.333	0.163	-0.168	0.393
Triglycerides (mmol/L)	-0.338	0.158	-0.145	0.460
Cholesterol (mmol/L)	-0.037	0.158	0.083	0.675
High-density lipoprotein (mmol/L)	0.281	0.243	-0.162	0.409
Low-density lipoprotein (mmol/L)	-0.172	0.481	-0.151	0.444
C-reactive protein (mg/l)	0.262	0.279	0.362	0.058
Ferritin (μg/L)	-0.035	0.888	0.005	0.979
Iron (μmol/L)	0.086	0.727	-0.258	0.186
Parathyroid hormone (pmol/l)	0.293	0.223	0.436	0.02
Calcium (mmol/L)	-0.319	0.182	0.087	0.659
Phosphorus (mmol/L)	-0.238	0.327	-0.027	0.892
Kt/v	-0.117	0.633	-0.305	0.115
Urea reduction rate	-0.214	0.378	-0.235	0.229
Body mass index (kg/m^2^)	-0.032	0.900	0.235	0.269

## DISCUSSION

This study showed that in patients on hemodialysis MAP was positively correlated with LS. Elevated LS was also correlated with CRP, PTH, and total bilirubin. Arterial pressure was the strongest predictor of LS value. To the best of our knowledge, this is the first study evaluating the impact of factors on LS assessed using RT 2D- SWE in this specific population.

Under physiological state, the liver receives one third of the blood flow through the hepatic artery and two-thirds through the portal vein. In advanced fibrosis, however, blood flow through hepatic artery increases (41). Our study results are in accordance with those by Piecha et al (25), who showed that, independent of the central venous and portal pressure, arterial pressure suffices to change LS. A meta-analysis by Bazerbachi et al (24) also confirmed the association between blood pressure and LS. It remains speculative whether this is due to increased arterial perfusion in chronic inflammation or due to elevated sinusoidal pressure, as it is represented in the sinusoidal hypothesis by Mueller et al (42). There also might be another unexplored mechanism contributing to LS elevation. Our results emphasize the importance of proper regulation of arterial blood pressure, although the exact pathophysiologic mechanism behind the association of MAP and LS observed in our study remains unknown. Therefore, more studies are needed for a better understanding of the physical parameters of LS and its adjustment depending on the arterial blood pressure.

Different factors can affect RT 2D-SWE detection of LS (43). As shown in our study, CRP significantly correlated with LS measurement, especially in patients with poor nutritional status (BMI<23). This is in accordance with previous studies exploring CRP value in CKD patients and its role in the pathogenesis of chronic inflammation, especially in the context of the malnutrition, inflammation, and atherosclerosis (MIA) syndrome (44). The central organ that produces and releases various classical inflammation biomarkers is the liver. Fibrotic liver increasingly produces diverse proinflammatory cytokines (45-48). Enhanced oxidative stress and release of inflammatory cytokines could create chronic inflammatory state and liver fibrosis (34). Furthermore, elevated CRP reflects the accumulation of pro-inflammatory cytokines in addition to being a strong risk factor for cardiovascular death (44). Our study revealed that the correlation between serum ALB and LS was negative but not significant. Similar findings were observed by Mikolasevic et al (34), but in HD patients with nonalcoholic fatty liver disease. In our view, our data indicate that in patients on HD, chronic inflammatory response interferes with the LS measurement in an unascertained way and elevates its value. It remains unclear whether the imbalance between proinflammatory and anti-inflammatory cytokines could somehow interfere with LS changes. However, high CR*P* values, low ALB, and increased LS are strong predictors of high mortality and probably interact with the MIA syndrome (34,44,49).

Previous reports have yielded heterogeneous results regarding the correlation of AST and ALT with LS. While Tapper et al (50) found a positive correlation between increased LS and ALT values, our findings are consistent with the findings by Shan et al (43), who found no significant positive correlation between liver enzymes and LS. Our observation can be explained by a decrease in the level of serum aminotransferases in CKD patients with a decline in kidney function. A possible explanation could also be related to pyridoxine deficiency or potentially to the existence of an unknown inhibitory factor in the uremic milieu, but the exact mechanism remains undiscovered (51-53). Our results indicate that in the assessment of LF in dialysis patients, serum aminotransferases are not a reliable marker of liver damage. In our study, total bilirubin positively correlated with LS, which is in accordance with the findings of other studies (32,33,54). This result demonstrates that intrahepatic cholestasis probably influences the LS value (55). We also showed that PTH was significantly positively associated with LS in the group with LS>7 kPa. According to Kirch et al (56), a liver function decline can impair the metabolic clearance of PTH fragments. This study indicates that elevated PTH in dialysis patients could be not solely a consequence of secondary hyperparathyroidism, but also partly of impaired liver function and consequently impaired metabolic clearance.

According to Cheng et al (28), lower PLT count was correlated with higher LS values. A possible explanation could be that PLT are regulated by thrombopoietin, which is produced by hepatocytes and the kidney. Thrombopoietin level is decreased in fibrosis, and consequently the PLT level declines (57,58). In our study, this finding did not reach statistical significance, probably due to different statistical methods or different inclusion criteria used. To reach definitive conclusions, there is a need for further studies with a larger number of participants.

The limitation of our study is the lack of liver biopsy performed in our patients, which prevented us from comparing LS values with histological characteristics.

In conclusion, our results show that arterial pressure affects LS. Moreover, LS should always be interpreted in combination with laboratory parameters. Further prospective studies are needed to clarify the pathophysiological pathway of arterial pressure affecting LS and to allow an insight into the pathogenesis of LS changes and their influencing factors in the hemodialysis population.
